# A Cross-Condition Single-Cell Atlas Defines Skeletal Muscle Adaptation and Its Predictive Limits

**DOI:** 10.3390/cimb48101080

**Published:** 2026-10-19

**Authors:** Wanting Tong, Renyu Yang, Qi Yu, Chunxue Shao, Guang Yang, Ziheng Wang

**Affiliations:** Division of Computational Biology, Chinese Center of Exercise Epidemiology, Northeast Normal University, Changchun 130024, China; tongwt077@nenu.edu.cn (W.T.); yangrenyu@nenu.edu.cn (R.Y.); yuqi123@nenu.edu.cn (Q.Y.); shaocx688@nenu.edu.cn (C.S.)

**Keywords:** skeletal muscle, single-cell RNA sequencing, single-nucleus RNA sequencing, muscle adaptation, cellular composition, spatial transcriptomics, predictive modelling

## Abstract

Skeletal muscle adapts to diverse stresses through coordinated changes across myofibres and cellular niches, yet these responses are usually studied separately. We assembled a cross-species discovery atlas of 501,940 cells and nuclei from six public resources, comprising 2863 condition–time–state combinations defined at the declared analysis unit level, and evaluated it with two independent validation datasets. Injury produced the largest observed compositional displacement, followed by mechanical overload, whereas acute human endurance exercise and denervation showed smaller occupancy shifts despite substantial cell-type-specific molecular responses. Genetic lineage data identified distinct newly fused myonuclear programmes; the overloaded Myomaker-knockout versus sham comparison remained limited by loading, genetic background and batch differences. Spatial analyses identified local enrichment of a terminal Schwann cell expression module around neuromuscular junction regions, and paired human datasets provided design-sensitive translational support. Across 20 fixed model specifications with training-only predictor preprocessing, the baseline-plus-context control model showed small average improvements over composition-only and intervention permutation controls, with heterogeneous performance across study holdouts. Here, we show that an evidence-graded atlas can characterise observed adaptive remodelling alongside study-dependent baseline structure while defining the limits of prospective prediction based on coarse composition.

## 1. Introduction

Skeletal muscle must preserve force production and metabolic homeostasis while repeatedly confronting demands that push the tissue in opposing directions. Exercise and mechanical loading promote oxidative remodelling, myonuclear accretion and growth, whereas denervation, inactivity and ageing favour atrophy and functional decline. Injury imposes a different problem: damaged fibres must be removed and rebuilt without allowing inflammation or extracellular matrix deposition to become persistent. These outcomes emerge from a tissue composed not only of multinucleated myofibres, but also muscle stem cells, fibro-adipogenic progenitors (FAPs), immune and vascular cells, pericytes, and cells associated with the neuromuscular junction (NMJ). FAPs support homeostasis and regeneration but can also generate ectopic adipose and fibrotic tissue, while coordinated monocyte–macrophage and regulatory T cell responses promote repair. Their responses are coordinated across time and space, making adaptation a property of the cellular ecosystem rather than of any single lineage [1,2,3,4,5,6,7,8,9].

Single-cell RNA sequencing (scRNA-seq) and single-nucleus RNA sequencing (snRNA-seq) have transformed the resolution at which this ecosystem can be observed. Foundational atlases established the diversity of adult muscle-resident populations and enabled comparisons across organs and ages [10,11,12]. Time-resolved studies have delineated myogenic progenitor states during regeneration, rare specialised myonuclear domains, inflammatory and senescent niches, and cell-type-specific responses to exercise, denervation and overload [13,14,15,16,17,18,19,20,21]. These studies establish that apparently similar tissue-level outcomes can be reached through distinct cellular programmes. Interpreting these states also requires distinguishing stress-associated expression from progression through myogenic programmes [16,22]. They also expose a conceptual limitation. Most cells or nuclei collected at different times are independent observations, not tracked descendants. A shift in the abundance of a state therefore measures tissue-level occupancy remodelling, not the probability that a particular baseline cell entered that state. Genetic lineage tracing, repeated biopsies and dense matched time courses provide stronger temporal evidence, but are much less common than cross-sectional snapshots.

This distinction matters because the field increasingly seeks to predict adaptation from the pre-intervention tissue. Baseline heterogeneity is biologically plausible as a determinant of future response, encompassing niche inflammation, myonuclear regulatory programmes and pre-existing stress-state variation [17,19,23]. Yet integrated single-cell analyses are highly vulnerable to study identity, species, sampling depth, muscle site and experimental design; integration must remove technical variation without erasing biological differences [24,25,26,27]. Large cell numbers do not compensate for a small number of animals or participants, and random cell-level splits can place observations from the same biological unit in both training and test sets. Likewise, differential-expression tests that treat cells as independent replicates can generate false discoveries [28,29,30,31]. A credible future-state atlas must therefore couple biological breadth to evidence grading, biological-unit-aware inference and explicit tests of cross-study transfer.

Existing muscle atlases provide essential components of such a framework but do not, individually, span the principal adaptive and maladaptive regimes. Regeneration atlases resolve injury and ageing, lineage studies distinguish newly fused from pre-existing myonuclei, exercise studies resolve acute regulatory responses, and spatial studies preserve tissue architecture [17,19,20,21,32]. What remains missing is a common quantitative coordinate system that compares how far the cellular ecosystem moves under different perturbations, distinguishes molecular response from abundance change, and asks which aspects of baseline structure survive stringent transfer tests. Importantly, a useful atlas need not claim direct single-cell ancestry where none was measured. It should instead state precisely which temporal estimand each design supports.

Here, we developed the Future Skeletal Muscle Adaptation Atlas by harmonising 501,940 cells and nuclei from mouse, rat and human muscle across injury and regeneration, overload, denervation, unloading and reloading, mechanotherapy, acute endurance exercise and population-scale resting muscle. We assigned Gold, Silver or Bronze evidence to each analysed component according to whether it provided lineage or within-subject information, a matched time series, or independent snapshots. We quantified future-state occupancy, compositional plasticity and entropy using declared analysis units based on animals, participants or conservatively combined captures. We then triangulated the atlas with genetic myonuclear lineage and fusion data, spatial NMJ analyses and paired human exercise datasets. Finally, we benchmarked 20 predictive model specifications under analysis-unit and study-level holdouts with permutation and nuisance controls. This design revealed an observed hierarchy of intervention-associated tissue remodelling and independently supported lineage and spatial adaptations, while showing that baseline composition alone does not provide a demonstrated, transferable, intervention-specific forecast of future muscle state.

## 2. Materials and Methods

### 2.1. Study Design and Evidence Tiers

Public datasets were registered before modelling with details of accession, species, assay, intervention, time, biological split unit and temporal-link type. Gold evidence comprised direct genetic lineage information and repeated sampling from the same human participant. Silver evidence comprised matched intervention and recovery or multi-time-point designs without direct lineage observation. Bronze evidence comprised independent baseline and post-intervention snapshots. The evidence tier constrained terminology but was not used as a statistical weight. The primary estimand was future-state occupancy conditional on the applicable observed baseline. It was not a same-cell transition probability.

The tiers are an operational classification of observed temporal linkage, not a calibrated confidence score, a universal evidence hierarchy, or a ranking by cell number, statistical significance or predictive accuracy. We applied the following decision sequence to the specific analysed component: (i) direct genetic myonuclear lineage information or verified pre/post samples from the same participant qualified as Gold; (ii) otherwise, a structured matched intervention time series or recovery design qualified as Silver; (iii) otherwise, independent baseline/post snapshots qualified as Bronze. A resource containing resting samples only was labelled Baseline, outside this temporal hierarchy. Computational label transfer, pseudotime, spatial proximity and model performance could not upgrade a tier. Gold lineage and Gold paired-subject evidence support different estimands: the latter observes a participant twice but does not track individual cells. Tier assignment did not remove small-sample, annotation or batch limitations. Table 1 records the applied criteria and claim boundaries for all eight analysed resources.

The analysed discovery resources were Zenodo record 19670721 (population-scale human baseline), GSE232106 (regeneration across lifespan), GSE294765 (overload, denervation and spatial NMJ profiling), GSE184413 (unloading, reloading and mechanotherapy), GSE183802 (denervation), and GSE240061 (paired acute endurance exercise and circadian control). GSE241035 supplied myonuclear lineage and fusion validation, and GSE270703 supplied paired human spatial training validation. Source-study designs and biological interpretation followed the corresponding publications [18,19,20,21,32,33,34,35].

### 2.2. Quality Control and Coarse Annotation

The revision analyses were rerun in R 4.6.1. Normalisation and harmonisation were guided by established count-model and variance-stabilisation principles [36,37]. For reprocessed scRNA-seq and snRNA-seq captures, library size, detected features and mitochondrial fraction were calculated within capture. Distribution-based lower and upper thresholds were estimated separately for each capture to avoid imposing a common threshold across assays. Doublets were called within capture using scDblFinder [38]. A cell or nucleus passed final quality control only if it passed the distribution filter and was classified as a singlet. For GSE183802, the primary reference and composition analysis retained all 29,539 publication-selected nuclei; local distribution/scDblFinder filtering was a separate QC diagnostic and was not used to redefine that reference. Published author-defined quality-control criteria were retained for GSE240061 and for the population-scale human baseline derived from the Finland–United States Investigation of NIDDM Genetics (FUSION) Tissue Biopsy Study (Zenodo record 19670721).

Published GSE183802 identities were mapped to raw barcodes and collapsed into coarse reference identities. The cross-study dictionary comprised 12 categories: myonuclei, NMJ myonuclei, muscle stem and myogenic cells, FAP and mesenchymal cells, endothelial cells, pericytes, smooth muscle cells, immune cells, Schwann or glial cells, adipocytes, tendon or myotendinous junction cells, and other or ambiguous cells. Cross-study labels were assigned using marker-based scores and reference transfer. Fine labels were collapsed only after within-dataset annotation. Leave-one-animal-out validation of the GSE183802 reference was performed by training on five animals and predicting the sixth.

### 2.3. Biological-Unit Harmonisation and Future Tasks

Captures were collapsed within the declared analysis unit, intervention point and time point before inference. For GSE294765, the unit identifier combines experiment and treatment/time (for example, Exp1 at three days after denervation), consolidating multiple captures conservatively; the resulting nine units, including seven future units, should not be read as independently verified individual animal identifiers. The primary task matrix retained the other/ambiguous category and the full 12-category dictionary, with zero entries for unobserved categories. It contained 40 future samples from 40 declared analysis units and 480 rows (40×12). We additionally performed an 11-category sensitivity analysis excluding other/ambiguous, renormalising each baseline and future composition before constructing the baseline mean. The ambiguous category is a measurement category, not a biological state. Zero counts were inserted for modelled states unobserved in a sample; these entries do not establish biological absence. Because cell-state occupancies are compositional, interpretation emphasised relative distributional change and biological unit replication [39,40]. For declared analysis unit *i* and state *k*, occupancy was

$$p_{ik}=\frac{n_{ik}}{\sum_{j}n_{ij}},$$

where $n_{ik}$ denotes the number of quality-controlled cells or nuclei assigned to state *k* in analysis unit *i*. For repeated-subject datasets, baseline occupancy was taken from the same participant’s pre-intervention sample. For other designs, it was the mean occupancy among observed species- and age-matched baseline units within the dataset. Where age was unspecified in the processed manifest, matching used the shared unspecified-age category; this does not verify exact chronological-age matching. Post-intervention units lacking the applicable baseline were excluded from primary future-state tasks. The five datasets with eligible future tasks contributed 20 regeneration, seven NMJ atlas, six acute exercise/circadian control, four disuse/recovery and three independent denervation samples. The resting human population resource supplied no future targets.

Composition plasticity was quantified by Jensen–Shannon (JS) divergence between baseline composition *p* and future composition *q*:

$$JS\left(p,q\right)=\frac{1}{2}D_{KL}\left(p∥m\right)+\frac{1}{2}D_{KL}\left(q∥m\right),\;m=\frac{p+q}{2},$$

using base-2 logarithms. Shannon entropy was $H\left(p\right)=-\sum_{k}p_{k}\log_{2}p_{k}$. These statistics describe distributional change and do not imply a transport coupling between individual cells.

### 2.4. Sensitivity to Study Inclusion, Baseline Construction and Annotation

We reconstructed the primary compositions from the saved sample-level counts, collapsing technical captures and checking the resulting 40 JS values against the archived dynamics table. For group-referenced designs, each future sample was compared separately with every eligible dataset-, species- and age-matched baseline unit. We also resampled complete baseline-unit composition vectors with replacement 2000 times within each matching stratum; each draw supplied a common baseline mean to all future units in that stratum. Future observations remained fixed. The 2.5th–97.5th percentiles of the resulting intervention medians are baseline-construction sensitivity ranges, not confidence intervals for an intervention effect. Pairwise baseline–baseline JS distances were retained as a heterogeneity diagnostic. Participant-specific baselines were never exchanged between people. Resampling a stratum with one baseline cannot estimate between-baseline variability.

The 500-cell rarefaction retained 39 of 40 future units; one circadian-control pair was ineligible, while all four exercise participants remained. Study-inclusion inventories restricted comparisons to mouse, snRNA-seq, or mouse snRNA-seq data and listed results after omitting each dataset in turn. These subsets remove already calculated within-study distances; they do not re-estimate an intervention effect or supply independent replications. We report only contrasts remaining observable in each subset; removing the sole injury study cannot test the injury ranking. Further checks excluded ambiguous assignments, required at least 2000 assigned cells per baseline and future unit, and raised annotation thresholds to a transfer score of 0.8 in GSE232106 and a marker-score margin of 0.3 in GSE184413 and GSE294765. The original transfer threshold was 0.5 and the original marker-margin ambiguity threshold was 0.15. These scores have different definitions and were not interpreted as comparable probabilities. Published labels were retained for GSE183802 and GSE240061. For the independent denervation cohort, we additionally excluded animal N2 (GSM5571588), whose leave-one-animal-out transfer accuracy was low, and assessed selective accuracy and retained coverage together. This deletion assesses the influence of that animal’s composition; it does not retrain the cross-study reference.

To examine capture-depth sensitivity, we sampled 500 assigned cells without replacement per eligible analysis unit composition after excluding ambiguous assignments, repeated this rarefaction 200 times, and reconstructed baselines in every repeat. Rarefaction quantifies finite-count sensitivity conditional on observed labels; it cannot remove assay-specific capture bias. Sensitivity analyses used seed 20261008. Complete unit-level results, inclusion counts and baseline eligibility are supplied in the Supplementary Reproducibility Package.

### 2.5. Generalisation Benchmark

Future occupancies and baseline occupancies were clipped to $[10^{-5},$ 1–$10^{-5}]$ before logit transformation. Time was converted to days and age to months; the time predictor was log(1+t). We evaluated 20 fixed specifications (Table 2). Hyperparameters were fixed rather than selected by validation score optimisation. The original neural network iteration cap of 300 produced non-convergence in 19 of 20 revised folds. We increased this numerical optimisation cap to 5000 for every fold; all 20 fits then reported convergence. This change was motivated by the training optimiser status, not by held-out accuracy; other candidate settings were retained. No cross-validated penalty selection, early stopping, inner tuning loop or nested model-selection procedure was used. The benchmark therefore compares fixed candidate specifications; choosing a winner retrospectively from outer-test performance does not produce an unbiased estimate of a selected model’s future performance.

For this revision we reran the benchmark with learned predictor preprocessing restricted to each training fold. Missing ages were replaced by the training-row median of observed ages (a fixed zero fallback was specified if none were observed); missing-age samples formed a separate unknown-age group for age-based splitting. Categorical vocabularies were learned from training data and represented by one indicator per observed training level. Unseen test categories received all-zero indicators for that variable, including its interaction terms; they were not reassigned to an observed category. No column was created from a test-only level. This convention prevents arbitrary reference category substitution but does not supply learned evidence for an unseen intervention or species. Count-derived occupancies, the harmonised state dictionary, unit conversions and clipping thresholds were fixed input definitions. Reference annotation itself was inherited from the atlas and was not retrained in each prediction fold. In particular, the GSE183802 reference supplied transferred labels for GSE232106; when GSE183802 was held out of occupancy-model fitting, its reference information still contributed to annotations in the training dataset GSE232106. Thus, predictor preprocessing and coefficient estimation were confined to training rows but the complete annotation pipeline was not independent of every held-out dataset. These are occupancy-model holdouts conditional on a shared representation, not end-to-end validation on an untouched study.

Penalised regression used training-only standardisation within glmnet [41]. Nearest-neighbour centring and standard deviations came from training rows only; zero or non-finite standard deviations were replaced by one. Ordinary least squares used a $10^{-7}$ diagonal stabiliser on non-intercept terms. Neural network inputs were not additionally standardised. The neural network and XGBoost [42] used the fixed settings in Table 2, with seed 20260715. All coefficient fitting used training responses only. The scripts expose each fold’s preprocessing audit.

There were 20 outer splits: five leave-dataset-out, eight leave-intervention-out, three leave-species-out, three leave-age-group-out (younger, older and unknown), and one greater-than-three-day extrapolation split. Known ages were divided at 12 months and time bins at three days. Every split was checked for disjoint dataset-qualified analysis unit identifiers; all composition rows from a unit stayed together. Inverse-logit predictions were obtained after limiting predicted logits to [−20,20] and then renormalised to sum to one within each future sample. Metrics were mean absolute error, root-mean-square error (RMSE), Spearman correlation, JS divergence and dominant-state accuracy. No cell-level random split was used.

Each specification was ranked by the unweighted mean of its mean JS divergence over all 20 outer splits; the eight lowest-scoring specifications were displayed. The heatmap shows the nine dataset, species and time-extrapolation splits, although selection uses all 20 splits. The per-dataset lowest-JS model is also reported descriptively. Splits overlap and are not independent replications. These rankings are exploratory and were not treated as a nested model-selection experiment.

### 2.6. Negative Controls, Variance Decomposition and Uncertainty

For each leave-dataset split, ridge models were compared with baseline-composition-only, future-capture-cell-count-only, study-identity-only and species-identity-only controls. Complete baseline-composition vectors were permuted between training analysis units, preserving cross-state covariance and each vector’s compositional sum; intervention and time labels were permuted at the training analysis unit level. Test labels were not permuted. Each permutation control used 25 replicates per held-out dataset. The observed model used baseline and design variables, excluding post-treatment expression, pseudotime and participant identity. The cell-count control used the total number of recovered cells in the observed future capture solely as a nuisance diagnostic; it is neither a sequencing-read-depth measurement nor a deployable baseline predictor. This total includes ambiguous assignments in both representation analyses, whereas the 11-category occupancy denominator excludes them. Each nuisance model included state identity plus only its named variable (baseline logit, future recovered-cell count, study or species); the restriction therefore refers to the information block beyond state identity. The observed control formula was baseline logit plus state, intervention, log time, age and species. Control ridge models used additive predictors, α=0 and fixed λ=0.1, with training-only predictor preprocessing. Permutation RMSEs were first averaged within each held-out dataset. Comparisons with the observed model report paired dataset-level RMSE differences, their range and the number of datasets showing improvement. Because training sets overlap between folds, their errors cannot be treated as independent for a fold-level sign-flip test. We therefore report descriptive comparisons without such significance tests. Figure error bars show standard deviation across the five dataset means, not across 125 independent studies.

In-sample variance was decomposed across baseline composition, state identity, intervention, time, species and age, and study. Shapley contributions were averaged exactly over all 6! orders in which covariate blocks entered a linear model. The residual was treated as unexplained variance, not labelled as biological stochasticity. Model-specification sensitivity was summarised as the standard deviation across the 20 predictions for the same held-out unit and state. Their empirical 5th–95th percentiles describe the spread of this fixed candidate set, not a calibrated predictive interval. Metric sensitivity ranges used 250 bootstrap resamples of complete held-out analysis units while keeping fitted models fixed; these conditional ranges do not propagate training, annotation or baseline-estimation uncertainty.

### 2.7. Differential Expression and Exact Small-Sample Inference

Differential expression was performed on biological-sample pseudobulk or sorted bulk-nuclear counts using DESeq2 [43]. Sample-level replication follows multi-sample benchmarks favouring replicate-aware pseudobulk inference; established alternatives include edgeR and MAST [29,30,44,45]. Cells or nuclei were not treated as independent replicates. Denervation composition was tested by enumerating all 63=20 possible three-versus-three animal assignments for both the 13 publication-defined labels and their 10 observed coarse categories. These source annotations do not separately identify Schwann/glial or smooth-muscle categories; we did not add identically zero tests for those two categories from the global 12-category dictionary. False discovery rates were calculated separately within each representation. Acute exercise composition was tested by enumerating all 64=15 possible assignments of four exercise and two circadian-control participants to the observed paired post-minus-pre statistic. These assignment tests require exchangeability of animal or participant labels under the null; enumeration alone does not establish randomisation or remove systematic group differences. Their minimum possible two-sided *P* values are 0.1 for the balanced denervation design and at least 1/15≈0.0667 for the exercise design. Paired spatial training sensitivity was assessed over all $2^{5}=32$ participant sign flips; its null requires sign symmetry of independent participant differences, or the corresponding randomisation justification. Its minimum two-sided *p* value is 0.0625. These small designs therefore cannot produce p<0.05 under the specified exact tests, even before multiple-testing correction. Multiple tests were corrected using the Benjamini–Hochberg procedure [46].

The retained denervation gene-level analysis aggregated counts by animal and published label, required at least 20 nuclei of that label in each of the six animals, and retained genes with at least five counts in at least three animals and at least 20 counts overall. Its design was ∼condition, with local dispersion-trend fitting and automatic outlier replacement disabled. The paired human spatial analysis summed QC-passing spots within each participant–condition section, retained genes with at least 10 counts in three sections, and used ∼subject+condition; the PH078-exclusion sensitivity retained the original gene set. These two archived analyses used the default independent-filtering target of 0.1 and were summarised at FDR below 0.05. Their fitted gene statistics are supplied as processed inputs rather than refitted in the compact package.

For GSE241035, count-level contrasts compared developmental green fluorescent protein (GFP)-positive and GFP-negative nuclei, overloaded newly fused GFP-positive and pre-existing GFP-negative nuclei, and overloaded Myomaker-knockout versus non-overloaded sham samples. The two GFP contrasts each comprised three paired biological preparations; positive and negative fractions shared the preparation identifier. Bulk-nuclear preparations pooled four gastrocnemius and four tibialis anterior muscles during development, or four plantaris muscles during adult overload, according to the source methods [20]. The paired comparisons used ∼preparation_pair+condition; the seven-sample knockout–sham comparison used ∼batch+condition. Genes with counts of at least 10 in at least two samples of the relevant contrast were retained. DESeq2 version 1.52.0 fitted the three contrasts with its default dispersion estimation and Wald test, with the independent-filtering target explicitly set to α=0.05; Benjamini–Hochberg adjustment was applied separately within each contrast. All coefficient fits converged, and all design matrices had full column rank. Wald confidence intervals and false discovery rate (FDR)-adjusted *p* values were reported. Sequencing libraries and individual sorted nuclei were not counted as additional biological replicates.

For knockout–sham sensitivity, we tabulated batch–condition overlap and checked the rank of the batch-plus-condition design before and after deleting the only sham in the knockout batch. Because the restricted batch contained three knockout preparations but only one sham preparation, we used a descriptive within-batch diagnostic instead of assessing within-batch differential expression. The knockout samples underwent overload, whereas sham samples did not; three sham preparations used the lineage reporter/Dox background and one used the conditional Myomaker/tamoxifen background [20]. Thus this contrast does not isolate fusion blockade from loading or genetic and induction context. Count size factors were median-of-ratios estimates from genes positive in all seven samples, normalised to geometric mean one. We compared mean $\log_{2}$(normalised count +1) in the three knockout samples with the single same-batch sham and repeated the comparison after omitting each knockout preparation, keeping size factors fixed. Agreement with the full-model DESeq2 effect direction is an internal stability check, not independent validation or a new FDR-controlled result.

### 2.8. Spatial NMJ Analysis

For each GSE294765 section, the terminal Schwann-cell score was the mean log(1+CPM) expression of available genes from *Plp1*, *S100b*, *Ngfr*, *Inhba*, *Nrxn1* and *Sox10*. Anchors were spots with a raw *Chrne* count greater than zero. Local spots lay within Euclidean distance four in array-row/array-column coordinates of any anchor; all remaining spots formed the far comparison. The observed local-minus-far score difference was compared with 500 random anchor sets of the same size on the fixed coordinates. The one-sided empirical *p* value was (1+#{null≥observed})/501. This null retains section geometry but does not explicitly preserve expression spatial autocorrelation or adjust for cell-type mixture. With two sections per condition, it is a localisation diagnostic, not evidence of condition-specific Schwann-cell activation or biological-replicate differential expression.

### 2.9. Software, Reproducibility and Reporting

Analyses were performed in R using SingleCellExperiment, Seurat, scDblFinder, DESeq2, data.table, glmnet, nnet, xgboost and ggplot2. Figures were generated in R and exported as 300-dpi PNG files for manuscript compilation. Model, control and conditional metric-bootstrap analyses used seed 20260715; baseline resampling, rarefaction and figure jitter used seed 20261008. The reconstruction analyses start from sample/state counts, fixed-threshold annotation-filter counts, held-out annotation predictions, task definitions, gene-collapsed integer counts for the 19 GSE241035 libraries, and processed spot scores. The GSE241035 contrasts are refitted using the declared paired or batch-adjusted designs. Denervation and paired-human spatial gene-level statistics are previously fitted inputs for count summaries and plotting; those gene-level fits, raw sequencing processing and annotation-model training are outside the reconstruction workflow. The annotation filter sensitivities use counts at fixed thresholds and do not support re-estimating thresholds from individual scores.

## 3. Results

### 3.1. An Evidence-Graded Atlas Spans Major Modes of Skeletal Muscle Adaptation

**Figure 1:**
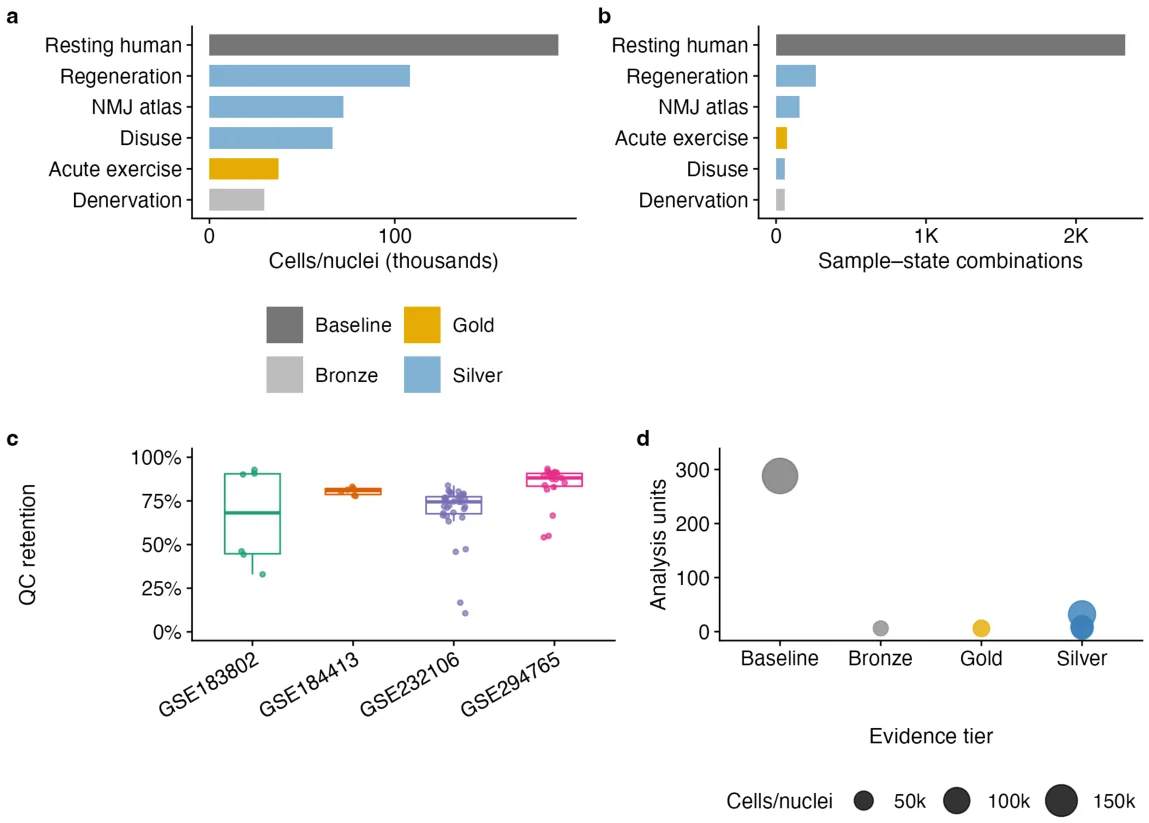
Atlas scope, evidence and quality control. (**a**) Analysed cells or nuclei for six integrated resources. (**b**) Positive-count sample–cell state combinations. (**c**) Capture-level final quality-control retention after distribution filtering and scDblFinder for four reprocessed cohorts. Points denote captures or libraries; boxes show median and interquartile range. GSE183802 local QC retention is a diagnostic; its primary reference uses the publication-selected set rather than this local-QC subset. (**d**) Evidence tier, declared analysis unit count and analysed cell count. Gold denotes paired human longitudinal or genetic lineage evidence, Silver denotes matched time-series designs and Bronze denotes independent snapshots. Cells and spots were never treated as inferential replicates.

We assembled six resources into a common cell state and analysis unit representation (Figure 1a,b) comprising 501,940 cells or nuclei, 2943 positive-count sample–state rows and 2863 unique analysis unit–condition–time–state combinations. The discovery atlas spanned population-scale resting human muscle, mouse regeneration across the lifespan, mouse overload and denervation with spatial profiling, rat unloading and recovery with or without mechanotherapy, independent mouse denervation, and paired human acute endurance exercise with circadian controls. Two additional datasets provided genetic lineage and paired human spatial validation and were excluded from the discovery cell-count threshold.

Evidence tier was separated from sample size: within-subject human biopsies and myonuclear lineage data were designated Gold, matched time series Silver, and independent snapshots Bronze. Snapshot comparisons were interpreted only as occupancy changes, not as lineage-resolved transitions.

Capture-aware quality control retained 108,192 of 163,080 regeneration cells, 66,439 of 82,900 disuse cells and 72,279 of 83,084 nuclei from the overload–denervation atlas (Figure 1c). All 29,539 nuclei in the independent denervation dataset were mapped to published identities and used for coarse reference annotation. Leave-one-animal-out validation achieved pooled accuracy of 0.876 and balanced accuracy of 0.853. Five animals achieved accuracies of 0.927–0.965; animal N2 (GSM5571588) achieved 0.485. For N2, 3187 of 4480 nuclei were predicted as other/ambiguous, including 1698 published myonuclei and 465 FAPs. In this validation the category derived from the reference *Unclassified* label and was predicted directly, rather than imposed by a low-score rejection rule. Its median prediction score was 0.957, showing that high transfer scores did not ensure correct assignment in this animal. Primary denervation compositions used the published identities rather than the held-out predictions; the transfer failure therefore tests reference portability, not a substitution of incorrect held-out labels into the primary denervation counts. Its influence and selective annotation coverage were examined explicitly below.

The population baseline contributed 188,337 resting human nuclei from 287 FUSION participants and one additional multiome sample, but temporal inference was restricted to datasets with observed age-matched baseline and post-intervention samples. The verified primary task matrix contained 12 composition categories, 40 future samples, 40 unique declared analysis units and 480 rows (40×12). Excluding other/ambiguous in sensitivity analyses left 11 categories and 440 rows. The six discovery resources retained their original total-cell accounting (Figure 1d).

### 3.2. Perturbations Show an Observed Ordering of Cellular-Ecosystem Displacement

**Figure 2:**
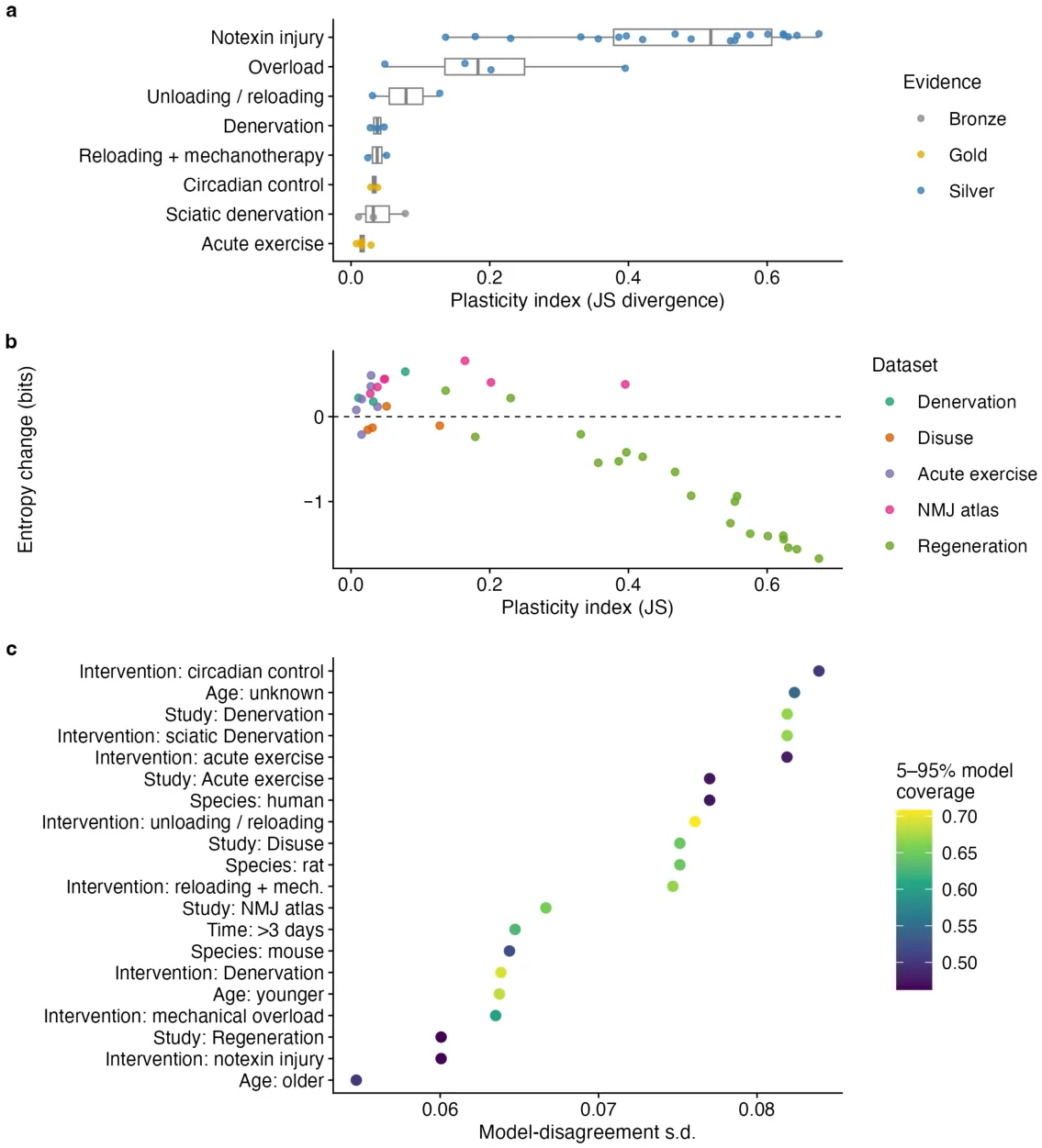
Occupancy plasticity, entropy and uncertainty. (**a**) JS divergence between the applicable baseline and future cell-state composition. Participant-specific pre-intervention baselines were used for repeated-subject designs; other datasets used dataset-, species- and age-matched baseline means. Points are declared analysis units; boxes show median and interquartile range. Gold, Silver and Bronze encode temporal evidence, not statistical significance. (**b**) Relationship between composition plasticity and Shannon-entropy change for each declared analysis unit. (**c**) Mean model-specification prediction standard deviation for strict held-out splits; colour denotes empirical coverage by the 5th–95th percentiles across 20 specifications. This is descriptive sensitivity to the chosen model set, not a calibrated predictive interval or a decomposition of epistemic and aleatoric uncertainty.

Future-state occupancy was defined as the fraction of each harmonised state within a post-intervention analysis unit. Comparisons used participant-specific baselines for repeated-subject designs and dataset-, species- and age-matched baseline means otherwise. Jensen–Shannon (JS) divergence quantified compositional displacement without implying cellular ancestry (Figure 2a).

Notexin injury produced the largest shift (median JS divergence, 0.519; interquartile range, 0.379–0.607; 20 biological units), often replacing endothelial-dominant baseline profiles with immune-dominant profiles. However, two geriatric baseline captures retained only 1472 and 956 cells after quality control. Injury was therefore the largest observed displacement, although sampling contributed to uncertainty in its magnitude.

Mechanical overload ranked next (median JS divergence, 0.183; interquartile range, 0.135–0.250; four declared analysis units). Median divergence was 0.079 after unloading and reloading and 0.038 during recovery with mechanotherapy. Denervation produced modest shifts in the NMJ atlas (0.038) and independent day-14 dataset (0.032). Acute endurance exercise showed a median divergence of 0.015 across four participants, compared with 0.033 in circadian controls. These small occupancy changes can coexist with molecular responses within a broadly stable coarse cell-state mixture.

Shannon-entropy changes captured a different aspect of composition from JS divergence (Figure 2b): analysis units could move far from baseline without becoming more even, or alter evenness with less displacement. Together, the measures distinguished distance from baseline from redistribution of state occupancy.

The broad ordering was retained under several sensitivity choices (Table 3). Excluding ambiguous assignments yielded median JS values of 0.529 for injury, 0.184 for overload, 0.039 and 0.011 for the two denervation cohorts, and 0.015 for acute exercise. Higher annotation thresholds yielded 0.518 for injury, 0.186 for overload and 0.058 for NMJ atlas denervation. Removing N2 changed independent denervation median JS from 0.032 to 0.044; after also excluding ambiguous assignments it was 0.029. Thus the broad separation of injury/overload from these smaller-displacement cohorts was retained, whereas the fine ordering among denervation, exercise and recovery conditions was sensitive to representation and cohort.

For N2, excluding ambiguous predictions and requiring transfer score at least 0.5 increased selective accuracy to 0.982 but retained only 1247 of 4480 nuclei (27.8%); a threshold of 0.8 retained 735 nuclei (16.4%) at an accuracy of 0.989. These gains cannot be interpreted as recovery of the full animal’s composition. Across the reannotated discovery cohorts, stricter filtering retained 98,157 of 108,192 regeneration cells, 55,886 of 66,439 disuse cells and 65,960 of 72,279 NMJ atlas nuclei. Confidence filtering can remove particular biological states disproportionately, and it cannot establish the correctness of all retained labels.

With 2000 complete-baseline resamples, the central 95% sensitivity range for median JS was 0.423–0.639 for injury, 0.124–0.285 for overload, 0.018–0.103 for NMJ atlas denervation and 0.011–0.085 for independent denervation. Baseline resampling was informative only for the four-baseline injury stratum, the two-baseline NMJ stratum and the three-baseline independent denervation stratum. Each age-matched disuse stratum and each paired human participant had one baseline, so a degenerate resampling range there does not imply baseline certainty. An average baseline and an individual baseline answer different questions: distance from a reference-group centre versus distance from one individual. Their JS values are not interchangeable, and group averaging can smooth baseline heterogeneity. Independent baseline–future contrasts contain between-animal and capture variability in addition to intervention-associated change, whereas a paired comparison controls stable participant differences but still contains biopsy and technical variation.

After rarefaction to 500 assigned cells, median intervention JS across 200 repeats was 0.524 for injury, 0.189 for overload, 0.041 for NMJ atlas denervation, 0.016 for independent denervation and 0.018 for acute exercise. Requiring at least 2000 assigned cells retained 17 injury future units and only one eligible injury baseline; injury median JS remained 0.569, compared with 0.167 for the three retained overload units. This restricted-baseline result is a sensitivity check, not an improved estimate with adequate baseline replication. Mouse-only comparisons retained injury above overload and denervation; snRNA-seq-only comparisons retained overload above denervation and acute exercise, but excluded injury entirely. Within GSE294765, overload exceeded denervation under the same species, muscle and assay, providing a less heterogeneous comparison. No available restriction simultaneously matches species, muscle, age, assay and sampling time while retaining every perturbation. Leave-dataset-out checks cannot validate a condition represented by the omitted study. The hierarchy therefore remains a description of the sampled cohorts, not a universal causal ranking of interventions.

### 3.3. Genetic Lineage Evidence Links Overload Adaptation to Newly Fused Myonuclei

**Figure 3:**
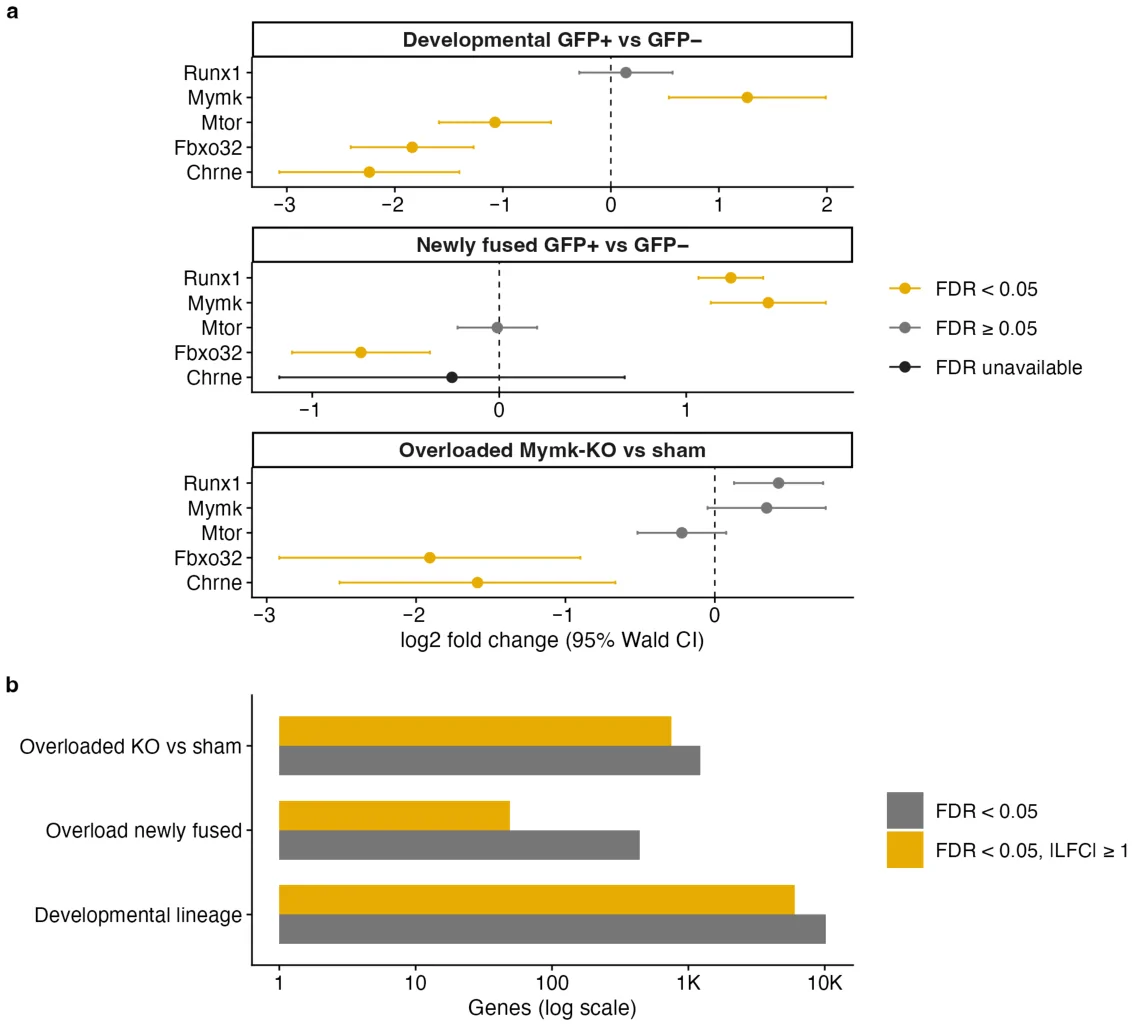
Paired genetic lineage and overloaded knockout–sham contrasts. (**a**) Selected gene effects from biological-preparation-level DESeq2 contrasts. Points show $\log_{2}$ fold change and lines show 95% Wald confidence intervals. Developmental and overload GFP-positive versus GFP-negative contrasts each used three paired pooled biological preparations with preparation blocking. Overloaded Myomaker-knockout versus non-overloaded sham used three versus four biological preparations with batch adjustment. Gold-coloured points denote FDR below 0.05; dark points indicate an unavailable FDR after independent filtering. (**b**) Numbers of FDR-significant genes and the subset with absolute $\log_{2}$ fold change of at least one. The knockout–sham design contained one sham in the knockout batch; its FDR values are conditional on the fitted model and cannot separate fusion effects from loading, genetic/induction background or limited batch overlap. Gene-level effects use the verified symbol-mapped table; Mymk and Runx1 were not FDR-significant in the knockout–sham contrast.

An independent genetic system distinguished newly fused GFP-positive myonuclei from pre-existing GFP-negative myonuclei during development and adult overload [20]. Reanalysis at the biological sample level yielded three complementary contrasts (Figure 3).

With preparation pairing included in the model, 10,173 genes differed between developmental GFP-positive and GFP-negative nuclei at a Benjamini–Hochberg false discovery rate (FDR) below 0.05; 6015 of these genes had an absolute $\log_{2}$ fold change of at least one. *Mymk*, which encodes Myomaker, was increased in newly fused developmental nuclei ($\log_{2}$ fold change 1.26, FDR $2.70\times 10^{-3}$). After adult overload, 441 genes differed between newly fused GFP-positive and pre-existing GFP-negative nuclei at FDR below 0.05; 49 of these genes had an absolute $\log_{2}$ fold change of at least one. The contrasts distinguish nuclear fractions in their respective contexts, but their DEG counts also depend on expression levels, dispersion and the small number of preparations and do not directly measure biological programme size.

We next compared Myomaker-knockout preparations subjected to overload with sham preparations without overload. The batch-adjusted knockout–sham contrast yielded 1219 FDR-significant genes, including 753 with an absolute $\log_{2}$ fold change of at least one. In the verified gene-symbol-mapped output, the knockout–sham $\log_{2}$ fold changes for *Mymk* and *Runx1* were 0.347 and 0.427, with FDR 0.627 and 0.100, respectively; neither met an FDR below 0.05 in this contrast. The gene-level values are shown in Figure 3a.

All three knockout samples were in batch 4, which contained only one sham (Sham4); the other three sham samples were in batch 3. The seven-sample batch-plus-condition design had rank three, but deleting Sham4 reduced it to rank two for three columns, making the treatment coefficient unidentifiable separately from the batch. Batch adjustment therefore cannot replace missing within-batch control replication. In a descriptive comparison restricted to batch 4, all 1219 full-model significant genes retained the direction of their adjusted effect, and 1212 retained that direction after each knockout sample was omitted in turn. The rank correlation between full-model adjusted effects and descriptive within-batch log-expression differences was 0.983 across tested genes. These results are expected to share information with the full-model contrast and are not an independent replication; every comparison still relies on the same single sham. Consequently, the 1219 changes are associations with the combined loading, genotype and induction context of this contrast, not an identified set of fusion-dependent effects. The sham preparations also differed in genetic reporter and induction background between batches, and the knockout group alone underwent overload. The direct paired GFP contrasts separately distinguish nuclear populations; identifying a fusion-specific response would require an appropriate comparison with fusion-intact overloaded controls and replicated conditions across compatible batches.

### 3.4. Molecular Responses Accompany Smaller Observed Composition Shifts

The distinction between molecular regulation and composition was clearest after denervation. In the day-14 dataset, exact enumeration of all 20 possible assignments of three control and three denervated animals produced no FDR-significant change among either the 13 publication-defined labels or their 10 observed coarse categories; their smallest observed unadjusted *P* values were 0.1 and 0.2, respectively. The smallest attainable two-sided permutation *P* value was 0.1 for either representation, so neither result can establish absence of abundance change. In contrast, animal-level pseudobulk differential expression identified extensive, cell-type-specific responses: 3169 FDR-significant genes in type IIB myonuclei, 567 in FAPs, 420 in type IIA myonuclei, 279 in type IIx myonuclei, 126 in macrophages and 124 in endothelial cells. The smaller observed composition distances therefore accompanied extensive transcriptional responses after loss of neural input; the exact composition test does not establish equivalence or preservation of cellular proportions.

A similar descriptive distinction appeared in paired human acute endurance exercise. Enumeration of all 15 possible assignments of four exercising and two circadian-control participants yielded an immune-cell exercise-minus-control difference in paired change of 0.0539, the largest estimate, with exact p=0.60; all six cell-type tests had FDRs 0.8. The minimum possible *p* value is at least 1/15≈0.0667, precluding significance at 0.05 for this design. These tests therefore cannot establish composition stability. The source study reported extensive cell-type and fibre-type regulatory circuitry after exercise [19]. At 3.5 h, those molecular responses coexisted with smaller observed coarse-composition distances in our reanalysis; a larger paired study is needed to quantify abundance effects precisely.

### 3.5. Spatial Analyses Localise a Schwann Cell Expression Module near the Neuromuscular Junction

**Figure 4:**
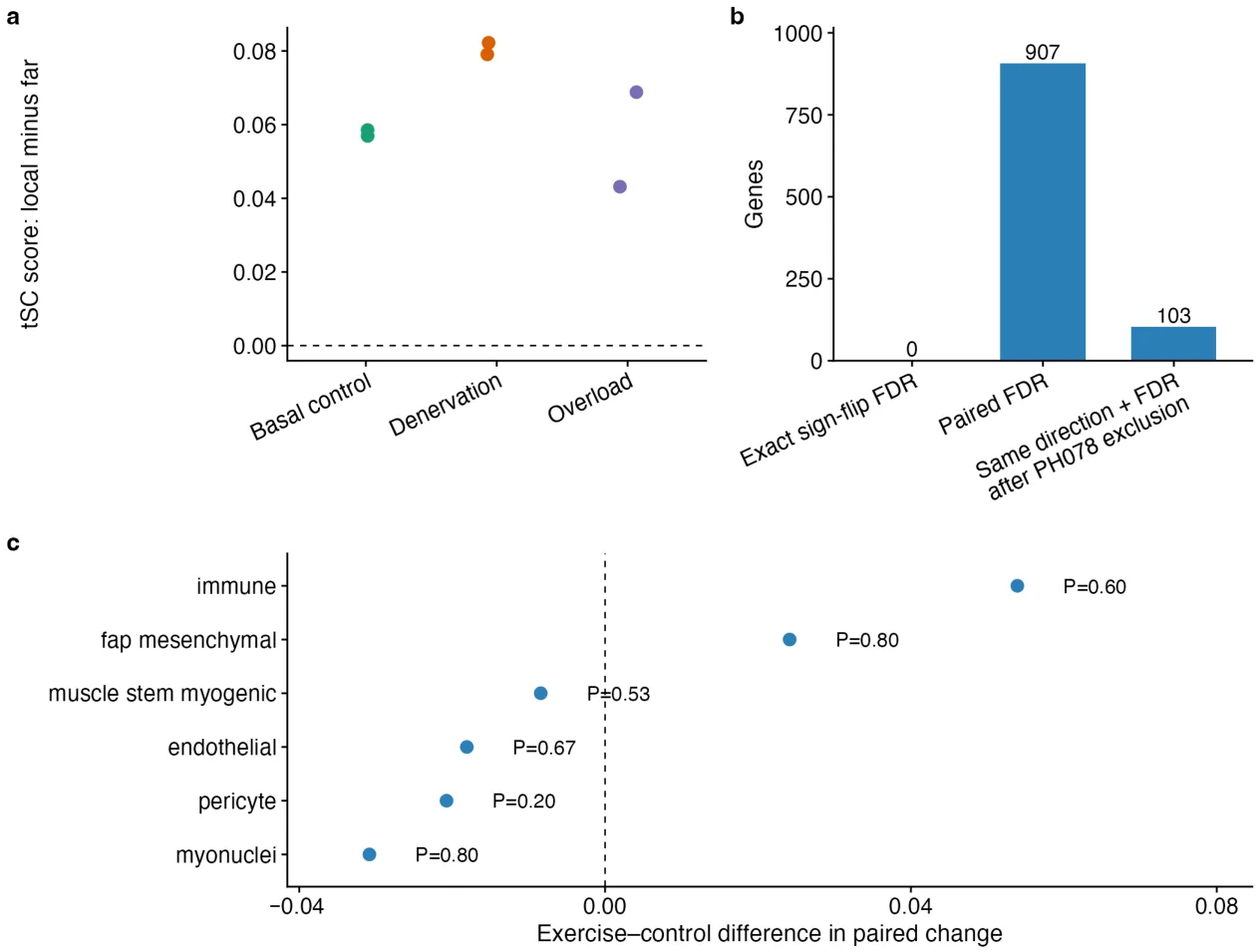
Spatial and paired-human validation. (**a**) Terminal Schwann cell score difference between spots local to *Chrne*-positive regions and far spots in six mouse sections. Within-section random-anchor *p* values are localisation diagnostics, not biological-replicate inference. (**b**) Paired human spatial pseudobulk results: 907 genes with paired-model FDRs below 0.05, 103 of those genes retaining both direction and FDR below 0.05 after PH078 exclusion (all 907 retained direction), and zero genes significant under exact five-participant sign-flip tests, whose minimum two-sided *p* value is 0.0625. (**c**) Exercise-minus-circadian-control difference in paired post-minus-pre composition change. *p* values enumerate all 15 possible four-versus-two participant assignments; all FDR values equal 0.8 and the minimum possible *p* value is at least 1/15, above 0.05.

We next asked whether a niche-associated response could be recovered in spatial data. In six mouse sections, terminal Schwann cell scores were higher near *Chrne*-positive NMJ regions than in far spots in every section (Figure 4a). Basal sections showed enrichment relative to the random-anchor diagnostic. The analysis was deliberately restricted to a spatial-null diagnostic because each condition contained only two sections; spots were not treated as independent biological replicates. This local enrichment is consistent with NMJ-associated Schwann cell expression, but does not establish activation changes between conditions; this interpretation is consistent with the multimodal source atlas [21].

Paired human spatial data provided a second translational layer. Whole-section pseudobulk modelling across five participants identified 907 genes with paired-model FDRs below 0.05 (Figure 4b). All 907 retained their estimated effect direction after exclusion of the low-quality PH078 pair, and 103 also retained FDRs below 0.05 in the four-participant refit. The loss of significance therefore does not mean reversal of the estimated effects; it can reflect reduced sample size, effect magnitude and dispersion estimation. No genes passed FDR correction of the exact sign-flip tests. With five paired differences, however, the minimum attainable two-sided sign-flip *p* value is $2/2^{5}=0.0625$, so significance at 0.05 is impossible even before multiple-testing correction. This resolution limit cannot independently refute the paired-model result. The data support directionally consistent model-based associations with sensitivity of significance to participant inclusion. These whole-section results do not establish cell-type-specific differential expression, but provide spatially preserved human evidence of training-associated transcriptomic differences. The source study further linked fibre-resolved changes to functional and morphological adaptation [32].

### 3.6. Study Holdouts Characterise the Limits of Baseline-Composition Prediction

We evaluated whether baseline composition could forecast future occupancy beyond structural regularities. Twenty fixed model specifications were rerun with training-only predictor preprocessing under leave-dataset-out, leave-intervention-out, leave-species-out, leave-age-group-out (including unknown age) and greater-than-three-day extrapolation splits. All splits were made by declared analysis unit; no random cell split was used. Specifications ranged from persistence and stratified means to linear interactions, ridge, lasso, elastic net, nearest neighbours, a shallow neural network and shallow gradient boosting.

**Figure 5:**
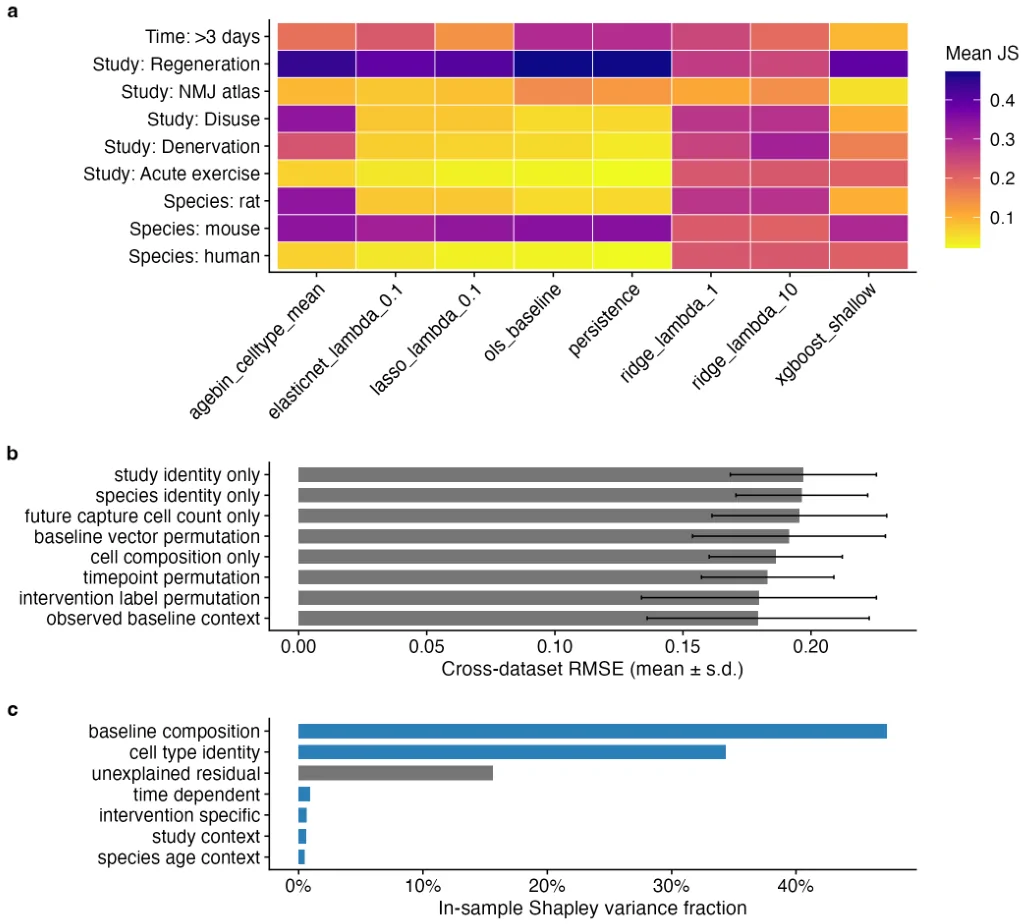
Study holdouts and negative controls. (**a**) Mean JS divergence for eight specifications selected by the lowest unweighted mean JS over all 20 outer splits; the nine leave-dataset-out, leave-species-out and greater-than-three-day splits are displayed. All 20 candidate specifications used fixed hyperparameters and training-only predictor preprocessing. Ranking is exploratory, not nested model selection; lower values are better. (**b**) Mean leave-dataset-out RMSE for observed and negative-control models; error bars show standard deviation across five held-out dataset means, with permutation replicates averaged first within each dataset. The observed baseline-context model had lower mean error, with heterogeneous dataset-specific differences; no independence-based fold-level significance test was applied. (**c**) Exact Shapley decomposition of in-sample target-logit $R^{2}$ across all 6! covariate-block orders. The decomposition is descriptive and is not evidence of cross-study predictability.

Several held-out datasets were described with low absolute errors by simple models (Figure 5a). Persistence performed best for human acute exercise (mean JS divergence 0.0224; root mean square error (RMSE) 0.0464; six participants) and for denervation (mean JS 0.0404; RMSE 0.0729; three animals). Baseline-only ordinary least squares performed best for disuse and mechanotherapy (mean JS 0.0557; RMSE 0.0528; four animals), whereas shallow gradient boosting performed best in the NMJ atlas (mean JS 0.0504; RMSE 0.0673; seven units). Regeneration was substantially harder: the converged five-hidden-unit neural network achieved mean JS 0.179 and RMSE 0.139 across 20 held-out units, with a dominant-state accuracy of 0.35. In these cohort-specific comparisons, persistence performed well where observed compositions changed less, whereas the injury cohort remained harder to predict. This association does not isolate perturbation magnitude from study design or establish a universal performance ordering.

Negative controls qualified the interpretation of cross-study performance (Figure 5b). The observed additive baseline-plus-context ridge model had a mean leave-dataset-out RMSE of 0.17938, versus 0.17968 after intervention permutation, 0.18307 after time permutation, 0.18621 for composition-only, 0.19138 for whole-baseline-vector permutation, 0.19542 for future-capture-cell-count-only, 0.19641 for species-only and 0.19700 for study-only. The observed model improved on composition-only in four of five datasets, with a mean RMSE advantage of 0.00684 and dataset-specific differences from −0.01367 to 0.03793. Relative to intervention permutation, its mean advantage was 0.00030, with improvement in three of five datasets and differences from −0.00535 to 0.00368. After excluding ambiguous assignments and rebuilding the 11-category baselines, observed-model RMSE was 0.19378 versus 0.20100 for composition-only and 0.19434 after intervention permutation. These are descriptive comparisons of five held-out studies with overlapping training sets, not independent significance tests. The small average advantage over intervention shuffling and heterogeneous study-level results motivate caution about intervention-specific transfer; they do not prove an absence of predictive information.

An exact Shapley decomposition over all 6! covariate-block orders attributed 47.3% of in-sample variance to baseline composition and 34.3% to cell-state identity (Figure 5c). Intervention, time, species and age, and study each accounted for less than 1%, leaving 15.6% unexplained. This decomposition was fitted in sample and is descriptive rather than evidence of transferable predictive information. Across held-out splits, mean prediction standard deviation ranged from 0.055 to 0.084, and the empirical 5th–95th percentile spread of model predictions contained 46–71% of observations (Figure 2c). The atlas therefore supports quantitative comparison of observed remodelling; individualised prospective prediction from composition alone remains unvalidated.

## 4. Discussion

The Future Skeletal Muscle Adaptation Atlas establishes a common, evidence-graded view of muscle remodelling across perturbations that are usually analysed separately. This ecosystem perspective is consistent with regeneration atlases resolving coordinated myogenic and intercellular programmes [13,14]. Its principal positive result is an observed cross-condition hierarchy of cellular-ecosystem displacement. Acute injury reorganised the tissue most strongly, mechanical overload produced an intermediate shift, and acute endurance exercise and denervation produced smaller coarse-composition changes. Because these conditions came from heterogeneous studies, this ordering is descriptive rather than a standardised causal comparison. The observed distance ordering can still inform biological comparisons: denervation showed thousands of cell-type-specific molecular changes alongside smaller composition distances, and acute exercise-associated regulatory circuitry coexisted with relatively small observed occupancy shifts. However, the exact denervation and exercise tests cannot reach p<0.05 at their current sample sizes, so their nonsignificant results are not evidence of unchanged composition. Future studies should therefore measure molecular state and occupancy as complementary axes rather than use one as a surrogate for the other.

The lineage and spatial analyses add molecular and tissue context to the abundance comparisons. Paired genetic separation of newly fused and pre-existing myonuclei identifies differences between nuclear fractions during development and adult overload. The overloaded knockout–sham contrast addresses a different question: it combines loading and genetic/induction differences with limited batch overlap and cannot identify fusion-specific regulation of resident nuclei. The source study examined additional control-overload contrasts and experiments beyond this reanalysis [20]. Enrichment of a terminal Schwann cell expression module near *Chrne*-positive regions provides localisation evidence in mouse muscle, but neither condition-specific activation nor cross-species conservation is established by this diagnostic. Such spatial context complements the specialised myonuclear domains resolved by single-nucleus profiling [15].

The human validations clarify the translational scale of these effects. Paired acute exercise data combined reported molecular responses with smaller observed occupancy distances at a single post-exercise time point; they cannot establish that molecular regulation precedes abundance change. Paired spatial training data showed broad model-based molecular associations with sensitivity of statistical significance to participant inclusion. These findings argue against defining exercise response from a single endpoint or molecular layer. Transcriptomic and multi-omic studies have similarly shown that training responses are distributed over time and across tissues [47,48]. A future predictive framework will require repeated measurements from participants, functional outcomes and harmonised baseline molecular features, rather than merely larger numbers of nuclei.

The generalisation analysis documents the performance and limitations of fixed occupancy models conditional on the atlas representation. Baseline composition explained substantial in-sample variance, whereas the observed model’s average improvement over intervention shuffling was small and its differences from controls varied across five held-out studies. This combination is compatible with dependence on stable study and design structure, but limited study-level replication prevents a definitive partition of transferable biology from such structure. It does not show that pre-existing biology is irrelevant. The direct genetic-lineage data identify expression differences between pre-existing and newly fused nuclear populations during overload. Instead, these data do not establish that coarse occupancy adequately represents the molecular, lineage and spatial states through which such effects may operate. Prospective prediction should therefore prioritise baseline gene-regulatory state, niche localisation, lineage information and standardised functional endpoints.

Several limitations define the scope of the resource. Public datasets differed in species, muscle type, age, sampling density and assay, and some conditions contained few independent biological units. Two geriatric baseline captures had severe quality-control loss, inflating uncertainty in injury-associated composition shifts. Collapsing dataset-specific labels into 11 named biological categories plus other/ambiguous increased cross-study comparability at the cost of fine substate resolution. The ambiguous category was retained in the archived primary analysis and explicitly removed in sensitivity analyses. Broad occupancy ordering persisted, but numerical distances and the ordering of smaller responses depended on annotation and baseline construction. Assay differences can alter myonuclear versus mononuclear recovery, muscles differ in fibre and niche composition, and species, age and sampling time are confounded with intervention. Most perturbations lack replication across independent matched studies; the primary injury comparisons also rely on the observed geriatric baseline stratum. Neither rarefaction nor restrictive inclusion removes these sources of confounding. Cross-study integration also entails a trade-off between batch correction and preservation of biological variation [26]. Evidence tiers describe temporal linkage only and must be read alongside these design-specific limitations. Snapshot data cannot identify same-cell transitions, residence times or continuous-time transition rates [49]. Likewise, disagreement across a fixed collection of specifications is not a calibrated uncertainty distribution and cannot separate epistemic from aleatoric variability. We therefore did not fit RNA-velocity, optimal-transport or continuous-time lineage models where the required spliced layers, dense matched time points or lineage links were absent [50,51,52].

Within these boundaries, the atlas organises half a million muscle cells and nuclei around intervention-aware, analysis-unit-level estimands, connects distributional remodelling to genetic, spatial and human evidence, and documents heterogeneous held-study performance of fixed occupancy models. Future forecasting studies should test whether baseline molecular and niche features improve prediction in prospectively collected cohorts, with annotation and predictor fitting separated from the validation data.

## Figures and Tables

**Table 1:** Operational evidence-tier assignments for the analysed components. A tier identifies the available temporal link; it does not certify statistical robustness or same-cell ancestry.

Resource	Tier	Observable Assignment Criterion	Supported Scope and Boundary
Zenodo 19670721	Baseline	Resting population samples without paired post-intervention outcomes	Baseline representation; excluded from future-occupancy targets.
GSE232106	Silver	Structured injury time course within age strata	Group-level occupancy changes; different animals, not tracked descendants.
GSE294765	Silver	Basal and matched 3/7-day perturbation sampling	Group-level temporal comparison; spatial sections provide localisation, not lineage.
GSE184413	Silver	Matched unloading/reloading and recovery design	Group-level recovery comparison; one observed baseline unit per age stratum limits resampling.
GSE183802	Bronze	Independent control and day-14 denervated animals	Cross-sectional occupancy and molecular contrasts; no within-animal pre/post link.
GSE240061	Gold, paired	Participant identifiers link pre/post biopsies	Within-subject acute response; neither same-cell tracking nor long-term adaptation.
GSE241035 (${\mathrm{GFP}}^{+}$/${\mathrm{GFP}}^{-}$ contrasts)	Gold, lineage	Direct genetic labels distinguish newly fused ${\mathrm{GFP}}^{+}$ and pre-existing ${\mathrm{GFP}}^{-}$ nuclei	Gold applies only to the paired ${\mathrm{GFP}}^{+}$/${\mathrm{GFP}}^{-}$ lineage contrasts. The separate overloaded Myomaker-knockout versus non-overloaded sham contrast is not assigned Gold lineage and is interpreted separately because loading, genotype/induction and batch differ.
GSE270703	Gold, paired	Five participant-linked pre/post spatial sample pairs	Within-subject training response; spots are not tracked cells or independent participants.

**Table 2:** Complete fixed candidate-model specification. *B* denotes baseline logit occupancy; *S*—state; *I*—intervention; *T*—log time; *A*—age in months; and *P*—species. Additive predictors are B+S+I+T+A+P; interaction predictors are B∗S+I∗S+T∗S+A+P, including main effects.

Model Family	Number	Settings and Prediction Rule
Persistence	1	Baseline occupancy carried forward.
Global and stratified means	6	Training target-logit means: global; state; intervention–state; time-bin–state; species–state; age-bin–state. Missing strata fall back to the state mean, then global mean.
Ordinary least squares	4	Baseline only; context (S+I+T+A+P); additive; interactions. Non-intercept numerical stabiliser $10^{-7}$.
Ridge	4	Interaction predictors; α=0, λ∈{0.01,0.1,1,10}, each a separate fixed specification.
Lasso	1	Interaction predictors; α=1, λ=0.1.
Elastic net	1	Interaction predictors; α=0.5, λ=0.1.
Nearest neighbours	1	Additive predictors; Euclidean distance after training-only standardisation; k=5 training state rows; arithmetic mean of their target logits.
Neural network	1	Additive predictors without explicit intercept column; five hidden units, linear output, decay 0.01, maximum 5000 iterations; fixed seed.
XGBoost	1	Additive predictors without intercept column; squared-error objective, depth 2, learning rate 0.05, row and column subsampling 0.8, 100 rounds, one thread; no early stopping.

**Table 3:** Median JS divergence under alternative composition and inclusion choices. Denervation columns distinguish GSE294765 (NMJ) and GSE183802 (day 14). Rarefaction entries are medians of intervention medians across 200 repeats. Complete counts and unit-level results are in the Supplementary Reproducibility Package.

Analysis	Injury	Overload	NMJ Den.	Day-14 Den.	Exercise
Primary, 12 categories	0.519	0.183	0.038	0.032	0.015
Exclude ambiguous	0.529	0.184	0.039	0.011	0.015
Higher annotation confidence	0.518	0.186	0.058	0.011	0.015
Exclude ambiguous and N2	0.529	0.184	0.039	0.029	0.015
Exclude ambiguous; minimum 2000 cells	0.569	0.167	0.039	0.011	0.018
Rarefy 500 assigned cells	0.524	0.189	0.041	0.016	0.018

## Data Availability

The public datasets analysed in this study are available from Zenodo under record 19670721 (https://doi.org/10.5281/zenodo.19670721 (accessed on 20 May 2026)) and from the NCBI Gene Expression Omnibus under accession numbers GSE232106, GSE294765, GSE184413, GSE183802, GSE240061, GSE241035, and GSE270703 (https://www.ncbi.nlm.nih.gov/geo/ (accessed on 20 May 2026)). No new biological specimens or raw sequencing data were generated in this study. The processed data and their provenance are publicly available at https://github.com/azusakou/muscle-state-occupancy-atlas (accessed on 20 May 2026). The analysis code, model specifications and dependency information are publicly available at https://github.com/azusakou/muscle-state-occupancy-atlas (accessed on 20 May 2026). The reconstruction workflow starts from processed inputs; its scope and limitations are documented in the Methods and repository README.
